# Iterative Pose Refinement for Object Pose Estimation Based on RGBD Data

**DOI:** 10.3390/s20154114

**Published:** 2020-07-24

**Authors:** Shao-Kang Huang, Chen-Chien Hsu, Wei-Yen Wang, Cheng-Hung Lin

**Affiliations:** Department of Electrical Engineering, National Taiwan Normal University, Taipei 106, Taiwan; grghwng@ntnu.edu.tw (S.-K.H.); wywang@ntnu.edu.tw (W.-Y.W.); brucelin@ntnu.edu.tw (C.-H.L.)

**Keywords:** object pose estimation, LINEMOD, deep learning, convolution neural network

## Abstract

Accurate estimation of 3D object pose is highly desirable in a wide range of applications, such as robotics and augmented reality. Although significant advancement has been made for pose estimation, there is room for further improvement. Recent pose estimation systems utilize an iterative refinement process to revise the predicted pose to obtain a better final output. However, such refinement process only takes account of geometric features for pose revision during the iteration. Motivated by this approach, this paper designs a novel iterative refinement process that deals with both color and geometric features for object pose refinement. Experiments show that the proposed method is able to reach 94.74% and 93.2% in ADD(-S) metric with only 2 iterations, outperforming the state-of-the-art methods on the LINEMOD and YCB-Video datasets, respectively.

## 1. Introduction

The pose of an object contains vital information to mimic how humans describe the position and orientation of real-world objects appeared in their vision. Making accurate estimate of the object pose favors not only robotic pick-and-place applications [1] but also augmented reality. The former adequately serves the rising need of factory automation in modern days, while the later integrates virtual objects in a real environment. As a result, object pose estimation has become a popular research topic over the past years. To allow steady and effective operations, six degree-of-freedom (DoF) object pose estimator focuses on predicting two matrices, i.e., the rotation matrix and the translation matrix, of a camera relative to a given object. The rotation matrix denotes the rotation between the camera coordinate system and the world coordinate system, whereas the translation matrix indicates the Euclidian distance between the origins of them. 

Traditional pose estimation methods can generally be divided into two categories: feature-based methods [2,3,4] and template-based methods [5,6,7]. The former detects candidate feature points in 2D image that are likely to be the projected positions of the 3D object model and then predicts the pose by matching the correspondence between 2D and 3D feature points. However, the feature-based methods are error-prone when handling texture-less objects. The latter, on the other hand, formulates each object and its pose status into a predefined template model with several parameters. By calculating the similarity between the captured image and the corresponding template with various candidate parameters, object pose can be estimated according to the parameter setting having the highest similarity score. However, one of the challenges of object pose estimation is that the scene condition lies in various aspects, such as object shapes, object texture, lighting conditions, objects in cluttered scenes, and occlusion between objects. For such reason, it is not easy to formulate a template that parameterizes such a complex situation. As a result, data-driven methods for object pose estimation have gained great popularity because of advancements of deep learning in recent years. 

Thanks to the rapid development of powerful graphical processing units (GPU), the data-driven techniques have made a great leap in pose estimation [8,9]. Recent methods [10,11,12,13,14,15,16,17,18,19,20,21,22] can be categorized based on the types of input data, i.e., RGB or RGBD. Traditional data-driven approaches [23,24] utilize convolution neural network (CNN) to select candidate feature points that roughly construct a bounding box surrounding the target object in 2D image, and subsequently solve the perspective-n-point (pnp) problem based on these points for pose estimation [25]. However, these methods are likely to encounter ambiguity of the pose estimation if the 2D–3D correspondences of the captured features are not accurate. Literatures [23,26] have suggested that adding an additional pose refinement process, such as the iterative close point (ICP) [27,28], is able to remedy this deficiency. Nevertheless, existing work [23] relying on ICP solution incurs a high execution time cost in the ICP calculation. Under such circumstance, the execution speed of other components in the method except ICP has to be further accelerated for increasing the speed of the entire pose estimation process. Instead of using ICP for refinement, DenseFusion [11] makes a rough pose estimate with CNN at first, and then concatenates an estimator network with an iterative refinement process. The refinement process has speed advantage compared with the time-consuming ICP. However, such refinement process in [11] only focuses on updating the geometry embedding for the pose refinement network during the iteration. The color embedding, on the other hand, remains intact during the entire refinement process. Thus, this deficiency motivated us to investigate whether modifying color embedding would be helpful to the iterative refinement process by utilizing the available data in the inner network. 

As an attempt to solve the above-mentioned problem, this paper proposes a pose estimation system which contains a pose estimator and a novel iterative refinement process, where the former roughly makes a pose estimate and the later revises the estimated pose to obtain better estimation result by iteratively updating the input data of the pose refinement network in both geometry and color. Because the refinement process uses basic image processing techniques, it is very simple for implementation. Although the pose refinement network mainly depends on geometry features, color features can be helpful for further improvement. Experimental results show that the performance of the proposed pose estimation system reaches 94.74% and 93.2% in accuracy with average distance of model points (ADD) metric, outperforming state-of-the-art methods on the LINEMOD [5,29] and YCB-Video datasets [23], respectively. 

The rest of the paper is organized are follows: Section 2 introduces the related work, Section 3 presents the proposed pose estimation method, Section 4 shows the training detail and experimental results, and the conclusion is given in Section 5.

## 2. Related Works

### 2.1. RGB-Based Pose Estimation

The entire pose estimation process [12] contains two components, i.e., object detection and pose estimation. The former localizes a target object and gathers available features of the target from the image, and the later estimates object pose by matching those features. PoseNet [30] combines the two components and regresses the pose by using CNN with a single RGB image. However, the lack of 2D–3D correspondence leads to difficulties in obtaining converged result for the regression. Since the pose data of an object contains 3D information, it is necessary to establish correspondence between the object model and the features gathered in the object detection stage for a concise representation. PoseCNN [23] feeds the captured image into CNN for extracting different task-specific features among various layers. Then, it generates semantic labels according to those features. Finally, the pose data are estimated by Hough voting and the regression based on the semantic labels. Unlike the approaches [23,30] that use regression to make pose estimate, keypoint-based methods [13,15,17] provide an alternative solution by using pnp solver. In the feature extraction stage, keypoint-based approaches estimate eight feature points in the captured image corresponding to the eight vertices of the bounding box of the object in 3D space. Then, pnp solver produces the final pose according to the 2D–3D correspondence. One drawback of such approaches is that the pose estimation error mostly relates to the mapping error between 2D and 3D key points. If the object in image encounters occlusion problem or cluttered background, 2D–3D localization is likely to be interfered, resulting in estimation error of the object pose. To deal with such problem, plenty of existing methods [13] focus on how to extract reliable object key points for pnp solver to make accurate pose estimate. PVNet [13] determines the key points with pixel-wise voting network to avoid estimation error under occlusion, and then utilizes uncertainty-driven pnp to estimate object pose. However, the key points in 3D object model must be predefined for such approaches. To sum up, the RGB-based pose estimation requires a great amount of data to compensate the lack of depth information. Otherwise, it requires a predefined 2D–3D correspondence in 3D space.

### 2.2. RGBD-Based Pose Estimation

Further robustness of pose estimation can be obtained with the availability of depth data. In fact, the depth of a target object is helpful to perform 3D localization and detect texture-less objects for pose estimation. For approaches [5,31] using depth map or point cloud, the correspondence between 2D pixel point and 3D point cloud can be easily established by using the available depth information. Note that the depth map can be transferred into point cloud format given the camera parameters. Some approaches [11,32] generate a candidate predicted pose and a corresponding confidence factor at each of the image patch region or data point subset of a point cloud through a CNN. The final pose estimate can be determined according to the confidence value. Tien [10] estimates rotation and translation separately to determine the final prediction by using uncertainty scores and RANSAC-based voting layer, respectively. DenseFusion [11] transfers image and depth map into high dimensional embeddings for each pixel, and then fuses these embedding data among patch regions in various scales. The fused data at each image patch region is fed into the estimator to generate a preliminary pose data. In the last stage, the pose is iteratively revised via an iterative refinement network. The input embedding data to the refinement network is iteratively updated based on the predicted pose. However, the refinement network in [11] only focuses on updating geometry embedding during the iteration. Hence, we are inspired to utilize the predicted pose to benefit both the geometry and color embeddings to obtain better performance. 

## 3. Methods

**Figure 1:**
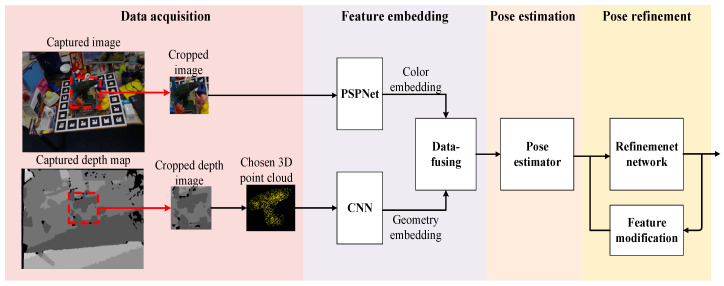
The architecture of the proposed pose estimation system.

Figure 1 shows the architecture of the proposed pose estimation system, where four stages are required for processing, including data acquisition, feature embedding, pose estimation, and pose refinement. The detail of each stage is described as follows.

### 3.1. Data Acquisition

When an image with 640 × 480 resolution is captured by the camera, object detection is applied to label the target object in the image. According to the detection result, we then generate a mask by applying the method in [23] where the region of interest (ROI) in the image is colored in white and the others are in black. After that, the captured image and depth map are processed separately based on the mask. We then crop the captured image to a smaller size according to the position and the size of the bounding box that fits the contour of the ROI. The bounding box is obtained by using open source OpenCV function “boundingReck”. On the other hand, the depth map is cropped into a smaller size based on the mask as well. Last, the cropped depth map and the pixel position of the mask in Cartesian coordinate system are transformed into point cloud data based on the camera parameters. Each pixel position (*x*, *y*) in the cropped depth map generates a 3D point (*X*, *Y*, *Z*) in the point cloud as follows:

(1) Z=d/s

(2) $$X=(x-c_{x})\cdot Z/f_{x}$$

(3) $$Y=(y-c_{y})\cdot Z/f_{y},$$

where d is the depth data at pixel position (*x*, *y*), *s* is the camera scale factor, and (*f_x_*, *f_y_*, *c_x_*, and *c_y_*) are the camera internal parameters.

### 3.2. Feature Embedding and 6D Pose Estimation

In this step, the features are gathered and described in a higher-level manner before being sent into the following pose estimator. Both the cropped image and the corresponding point cloud data are separately taken as the inputs of two independent neural networks to generate color embedding and geometry embedding, respectively. The cropped image is normalized and then fed into a Pyramid Scene Parsing Network (PSPNet) [33] for generating semantic high dimensional features, whereas the cropped point cloud is fed into a CNN for feature-sensing in various sizes of local region. Next, the local and the global features are jointly fused at each pixel according to the above two types of embedding data. Thus, this process produces a high dimensional data at each pixel that makes it named as “dense data.”

In the pose estimation stage, the rotation matrix and the translation matrix are estimated in this step based on the dense data. In the forward process of propagation, the input dense data pass through a CNN to generate a quaternion rotation matrix, a translation matrix, and a confidence value given the target object. Since we built our method upon DenseFusion [11], we use the same component in this stage.

### 3.3. Pose Refinement

**Figure 2:**
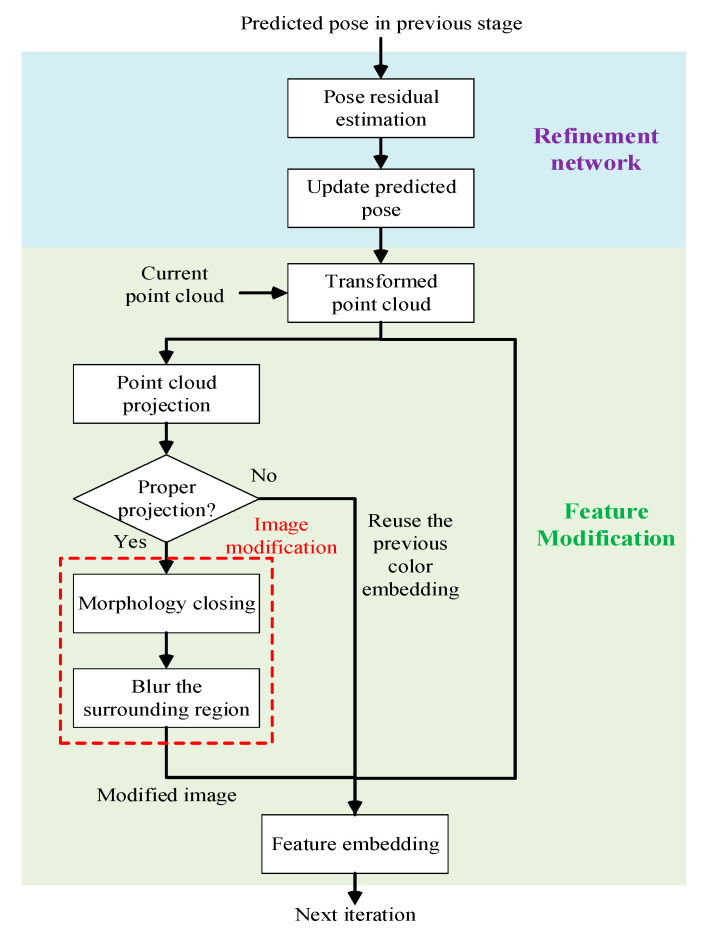
The proposed iterative pose refinement process.

The stage of pose refinement is an iterative refinement process which learns how the pose is gradually varied to narrow the gap between the prediction and ground truth. In fact, the residual of the predicted pose is learned by iteratively setting the current transformed point cloud as the new input data of the refinement network. That is to say, the geometry embedding is changed based on the predicted pose during the iterative process. Compared with the structure of DenseFusion [11], we extend the use of the predicted pose for improving the color embedding. The architecture of the proposed iterative pose refinement process is shown in Figure 2, where the estimated pose in the previous stage is fed into a pose residual estimation network at the beginning. Then, the predicted pose is updated and then utilized to transform the current point cloud for revising the geometry embedding and the color embedding. The reconstruction of geometry embedding is a straightforward decision because the refinement network has to know the change of geometric structure of the point cloud based on the predicted pose. However, the revision of color embedding requires a judgment as to when we should launch the revision in the refinement process. Once the process has launched, we project the chosen point cloud onto the captured image by using the predicted pose and the camera internal parameters, and then count the amount of the projected points which lie in the bounding box region estimated from the previous data acquisition stage. Here, we compute the following score *f* as

(4) $$f=n_{i}/n,$$

where *n* is the total number of the pixel positions projected from the chosen point cloud, and *n_i_* is the number of the projected points which lie within the bounding box region. If *f* is greater than 0.8, we launch the following image modification and embed the modified image into color embedding at the last step of the refinement process. Otherwise, the previous color embedding is used for the next iteration. 

**Figure 3:**
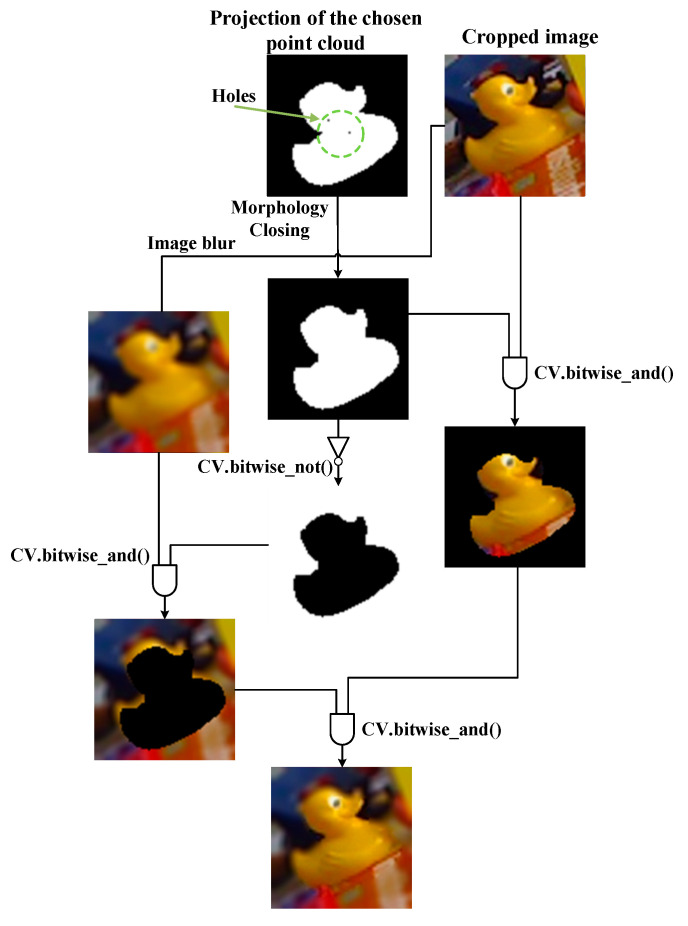
The process of image modification.

The image modification is a process that blurs the entire region except the ROI. The purpose of this modification is to reduce the redundant factor of the color embedding. This is because the color embedding of the cropped image is a high-dimensional vector containing the gradient of color at each pixel position. If we blur the surrounding region of ROI, the amount of the redundant gradient can be suppressed in the feature embedding process, resulting in a better pose refinement performance. To accomplish the task, image processing is applied to the cropped image. Figure 3 shows the entire image modification process. When the chosen point cloud is projected on the image, a binary image is generated where the projected positions in image are colored in white and the others are in black. However, there are holes exist in the generated binary image. Hence, we apply morphology closing to deal with the holes to generate a binary mask *I_b_* indicating the current pose projection. The resulting image *I_o_* is

(5) $$I_{o}=(I_{c}\wedge I_{b})\wedge (G(I_{c})\wedge \bar{I_{b}})$$

where *I_c_* is the cropped image, “∧” is the AND operator, and *G*(*x*) is a two dimensional Gaussian blur function with zero mean. The logical AND and NOT operators are implemented with OpenCV open source functions “CV.bitwise_and” and “CV.bitwise_not”. Although there is a binary mask generated in the data acquisition stage, it is our goal to utilize the available source in the inner network and maximize the use of the predicted pose for improving the performance of the pose refinement process.

## 4. Experimental Results

In order to evaluate the proposed pose estimation system, we conduct our experiments on Intel (R) Core(TM) i7-7700 @ 3.6GHz and a NVIDIA GeForce GTX 1080 graphic card. The well-known LINEMOD and YCB-Video datasets [23] are chosen for the evaluation of 6DoF pose estimation. During the training process, the ground-truth mask of each scene is used in the data acquisition stage. Note that the ground-truth mask can be generated by projecting the point cloud of the object onto the image plane based on the ground-truth pose. The critical part of the training process is to decide when to launch the image modification process, because such process highly depends on the performance of the predicted pose. If the predicted pose is inaccurate that cannot make proper 2D projection, the image modification will blur the ROI and then formulate an inaccurate color embedding for pose estimation, making the training process difficult to converge. Hence, we launch the image modification only when the error of the predicted pose is small enough. In fact, we manually select the modification launch time when the learning curve is becoming saturated within a steady margin during the training process.

There are two metrics for performance evaluation in this paper, i.e., the average distance of model points (ADD) [11] and the area under curve (AUC) [22]. Note that the symmetric objects will be handled with ADD-S [11], which indicates ADD metric for symmetric objects. Table 1 shows the accuracy in ADD(-S) metric of the proposed method, in comparison with the state-of-the-art approaches, including BB8 [15], SSD-6D [16], PVNet [13], Tien [10], and DenseFusion [11]. We take the best performance record according to the original papers. In addition, the iterative refinement process of the proposed method is only executed two times for a fair comparison with DenseFusion. In Table 1, we can see that the average accuracy of ADD(-S) metric reaches 94.74%, outperforming the state-of-the-art methods. Note that the average accuracy in ADD(-S) metric of the proposed method is also better than DenseFusion if we increase the number of iterations to four. That is to say, based on similar system architecture, the image modification process is helpful for improving the iterative refinement network. In order to prove the validity, we utilize the same trained parameters of DenseFusion to execute the proposed method on the YCB-Video dataset for evaluation. We follow the same AUC setting in [23] and set the threshold of the ADD-S to 0.1 m. Table 2 shows the accuracy of AUC test in ADD-S metric of the proposed method, in comparison with Tien [10], PoseCNN+ICP [23], and DenseFusion. We can see that the average AUC metric of the proposed method still slightly out-performs the state-of-the-art methods. 

**Figure 4:**
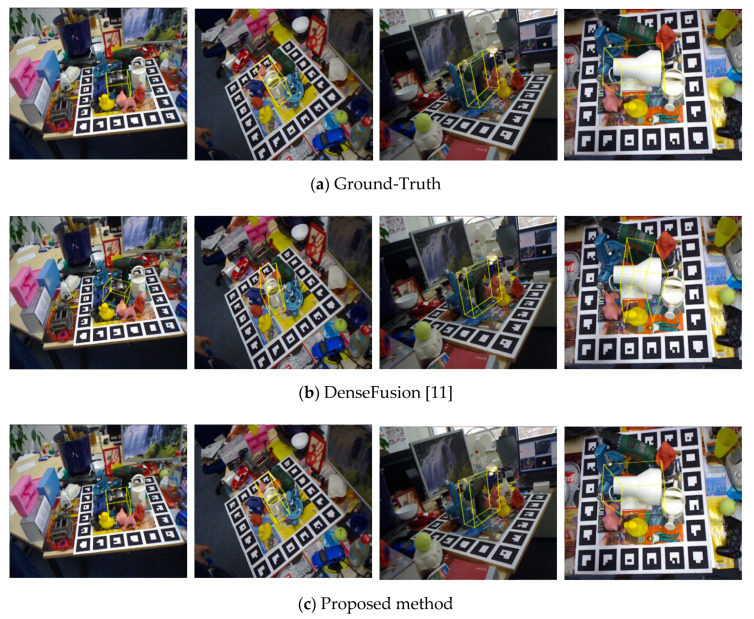
The object pose estimation results by yellow bounding box are drawn in the corresponding scene images according to the LINEMOD dataset. The name of the target objects in column from left to right are Cam, Can, Driller, and Lamp, respectively.

**Figure 5:**
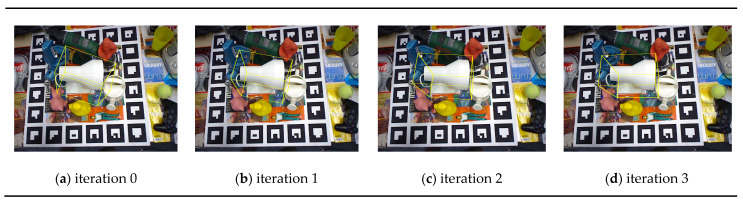
The evolving process of the estimated pose during the refinement process, for the scene shown in the far right column in Figure 4.

Figure 4 shows some of the pose estimation results on the LINEMOD dataset compared with DenseFusion, where the pose refinement process of both the proposed method and DenseFusion is executed for four iterations for a fair comparison. In Figure 4, the far-left column of the figures shows that the target object ”Cam” is occluded by the surrounding objects, resulting in failure for DenseFusion to provide an accurate estimate. On the contrary, the proposed method is able to make an accurate estimate in such condition. Moreover, according to the scene of the far right figures in column in Figure 4, we can see that the target object “Lamp” is an object colored in white, while the neighboring object “Can”, shares the same color. Hence, the gradient information of both two objects is mixed together in the color embedding data at the overlapped region between the two objects in the image. In this scene, DenseFusion makes an incorrect pose estimate while the propose method can make a satisfactory estimate of the object pose closer to the ground-truth. The evolving process of the refinement network for the same scene is shown in Figure 5, where the proposed method can gradually correct the pose and iteratively derive the final pose closer to the ground truth even the initial pose estimate is incorrect.

**Figure 6:**
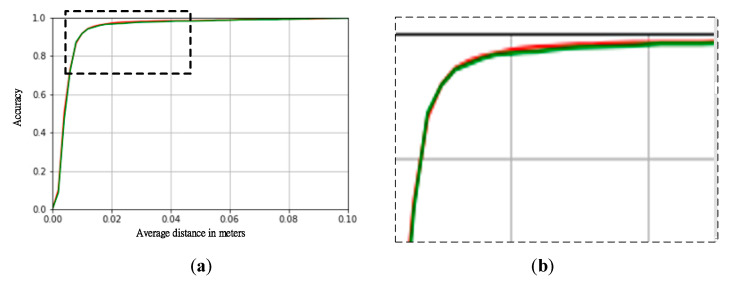
(**a**) The accuracy-threshold curves of DenseFusion [11] (green line) and the proposed method (red line) based on LINEMOD. (**b**) A closer look of the enlarged portion indicated by the black-dash rectangle in (a).

In the test of AUC on the LINEMOD dataset, the proposed method reaches 94.79%, which is slightly better than 94.49% of DenseFusion. According to the experiment in [22], we plot the accuracy-threshold curves of DenseFusion and the proposed method as shown in Figure 6. We can see that although the two curves are almost overlapped, the red line of the proposed method is slightly higher than the green line of DenseFusion when the threshold is larger than 0.02. Thus, the accuracy of the proposed method is still better than DenseFusion even the threshold setting becomes larger.

The advantage of the proposed method is that we improve the pose refinement process by adding an image modification based on both geometric and color components. In addition, the image modification step of the proposed method includes only basic openCV functions which bear a low computational cost. Specifically, the execution time of the image modification step is around 24 ms per frame. We believe that there is room for improvement if the codes can be executed on GPU. There are, however, the limitations of the proposed method, which lie in the 2D projection process and the parameter setting of mask-generating in the image modification step. The former indicates that the initial estimated pose has to make a proper 3D-to-2D projection close to the ROI region for the following mask-generating process. Otherwise, the blurring process will incur negative impacts to the generated color embedding since the important color features are suppressed. Moreover, 2D projection process needs a careful selection of the launch time of the image modification in the training process. The later indicates the need of an adaptive kernel size selection for generating the mask in the pose refinement process. If the projected point cloud is not sufficiently dense enough, the kernel size of morphology opening has to be adaptively increased for hole filling process. It is our plan to remove these limitations by adopting learning techniques in the future.

## 5. Conclusions

We developed a novel iterative pose refinement process that utilizes the predicted pose to update both the color and geometric embedding for obtaining better performance on object pose estimation. Since the color embedding for pose estimation contains high-dimensional information extracted at the local region of an image, our method aims at reducing the image redundant factor so that the embedding data can suppress the interference of surrounding objects. Thus, the proposed method can reduce estimation error when the color of surrounding objects is similar to that of the target object. Experimental results show that the proposed method outperforms the state-of-the-art DenseFusion method in both ADD and AUC metrics. Moreover, the design of the proposed refinement network only utilizes the available data of the inner network. This makes the proposed method flexible to support other designs for object pose estimation. 

## Figures and Tables

**Table 1:** Comparison between the proposed method and state-of-the-art approaches on LINEMOD in ADD(-S) metric, where the best results are marked in bold. Note that * denotes the objects are symmetric.

	BB8 w/Ref. [15]	SSD-6D w/Ref. [16]	PVNet [13]	Tien [10]	DenseFusion [11]	Proposed Method
Ape	40.4	65	43.62	85.03	92	92.95
Bench vise	91.8	80	**99.90**	95.54	93	92.05
Cam	55.7	78	86.86	91.27	94	**96.96**
Can	64.1	86	**95.47**	95.18	93	93.31
Cat	62.6	70	79.34	93.61	**97**	96.31
Driller	74.4	73	**96.43**	82.56	87	88.80
Duck	44.3	66	52.58	88.08	92	**92.95**
Eggbox *	57.8	**100**	99.15	99.90	**100**	99.71
Glue *	41.2	**100**	95.66	99.61	**100**	99.90
Hole pucher	67.2	49	81.92	**92.58**	92	91.15
Iron	84.7	78	**98.88**	95.91	97	96.32
Lamp	76.5	73	**99.33**	94.43	95	94.91
Phone	54.0	79	92.41	93.56	93	**96.34**
Average	62.7	79	86.27	92.87	94	**94.74**

**Table 2:** Comparison between the proposed method and state-of-the-art approaches on YCB-Video dataset in AUC metric, where the best results are marked in bold.

	Tien [10]	Posecnn+ICP [23]	DenseFusion [11]	Proposed Method
002_master_chef_can	93.9	95.8	**96.4**	**96.4**
003_cracker_box	92.9	91.8	95.5	**95.8**
004_sugar_box	95.4	98.2	97.5	97.6
005_tomato_soup_can	93.3	94.5	**94.6**	94.5
006_mustard_bottle	95.4	98.4	97.2	97.4
007_tuna_fish_can	94.9	97.1	96.6	**97.1**
008_pudding_box	94.0	97.9	96.5	**96.0**
009_gelatin_box	97.6	**98.8**	98.1	98.0
010_potted_meat_can	90.6	**92.8**	91.3	90. 7
011_banana	91.7	**96.9**	96.6	96.1
019_pitcher_base	93.1	**97.8**	97.1	97.5
021_bleach_cleanser	93.4	**96.8**	95.8	95.9
024_bowl	**92.9**	78.3	88.2	89.5
025_mug	96.1	95.1	**97.1**	96.7
035_power_drill	93.3	**98.0**	96.0	96.1
036_wood_block	87.6	90.5	89.7	**92.8**
037_scissors	**95.7**	92.2	95.2	92.1
040_large_marker	95.6	97.2	97.5	**97.6**
051_large_clamp	**75.4**	**75.4**	72.9	72.5
052_extra_large_clamp	**73.0**	65.3	69.8	70.0
061_foam_brick	**94.2**	97.1	92.5	92.0
Average	91.8	93.0	93.1	**93.2**
